# Ozonation of Selected Pharmaceutical and Personal Care Products in Secondary Effluent—Degradation Kinetics and Environmental Assessment

**DOI:** 10.3390/toxics10120765

**Published:** 2022-12-08

**Authors:** Fátima Jesus, Eva Domingues, Carla Bernardo, Joana L. Pereira, Rui C. Martins, João Gomes

**Affiliations:** 1 Department of Environment and Planning, CESAM—Centre for Environmental and Marine Studies, University of Aveiro, Campus de Santiago, 3810-193 Aveiro, Portugal; 2 CIEPQPF—Chemical Engineering Processes and Forest Products Research Center, Department of Chemical Engineering, Faculty of Sciences and Technology, University of Coimbra, Rua Sílvio Lima, 3030-790 Coimbra, Portugal; 3 Department of Biology, CESAM—Centre for Environmental and Marine Studies, University of Aveiro, Campus de Santiago, 3810-193 Aveiro, Portugal

**Keywords:** ozone, advanced oxidation process, ecotoxicity assessment, aquatic systems, wastewater treatment plants

## Abstract

The efficiency of ozonation depends on the water matrix and the reaction time. Herein, these factors were addressed by assessing the removal of five pharmaceutical and personal care products (PPCPs) by ozonation. The main aims were: (i) to assess the effects of the water matrix on the degradation kinetics of PPCPs, individually and in mixture, following ozonation; and (ii) to assess the ecotoxicological impact of the ozone reaction time on the treatment of a spiked municipal wastewater (MW) added the five PPCPs over several species. The degradation of the PPCPs was faster in ultrapure water, with all PPCPs being removed in 20 min, whereas in the MW, a 30 min ozonation period was required to achieve a removal close to 100%. Increasing the number of PPCPs in the water matrix did not affect the time required for their removal in the MW. Regarding the ecotoxicity assessment, *Raphidocelis subcapitata* and *Daphnia magna* were the least sensitive species, whereas *Lemna minor* was the most sensitive. The temporal variation of the observed effects corroborates the degradation of the added PPCPs and the formation of toxic degradation by-products. The removal of the parent compounds did not guarantee decreased hazardous potential to biological species.

## 1. Introduction

Water scarcity is currently a great concern and acute problem to solve in a large number of countries [1]. In particular, some of these countries are facing green water scarcity, which represents a condition where the rainwater cannot fulfill the crop water requirements [2]. Therefore, it urges the need to reuse water from municipal wastewater treatment plants (MWTPs), at least for agriculture irrigation [3]. Indeed, this is already the most common end use of treated wastewater in many countries [3,4]. However, the reuse of such reclaimed water for crop irrigation might impact environmental and human health due to the presence of toxic compounds [3,4]. Moreover, modern society relies heavily on the use of chemical compounds, namely pharmaceuticals and personal care products (PPCPs), additives in food and textiles, and home appliances, among others. This leads to wastewater being increasingly polluted with a high variety of chemical compounds. Hence, water resources are not only becoming increasingly scarce, but also increasingly polluted.

MWTPs receive a huge variety of contaminants of emerging concern (CECs), such as flame retardants, pesticides and PPCPs [5]. Some of these PPCPs, such as the pharmaceuticals carbamazepine (CBZ), sulfamethoxazole (SMX) and paracetamol (PCT) are released to the municipal wastewater because they are not completely absorbed by the human body [6,7]. For instance, around 58–68% of unchanged PCT (the most popular analgesic, also known as acetaminophen) is largely excreted by the human body and it has a low level of removal in MWTPs [7,8]. Other problematic CECs are the parabens, which are often used as antimicrobial and preservative agents in cosmetics, pharmaceuticals and food and beverage processing [9]. These compounds have also been detected in surface water, influents and effluents of MWTPs, as well as in sewage sludge [10]. Moreover, the above-mentioned PPCPs have been detected in water resources at the micro and nanogram scale [10,11,12,13]. Conventional biological effluent treatments are not efficient to remove these PPCPs since they are not adsorbed or biodegraded by active sludge, owing in some cases to their polarity and antibacterial nature [14]. For instance, SMX concentrations of 24.8 μg L^−1^ have been reported in a wastewater treatment plant secondary effluent [14]. Ribeiro and Pedrosa [15] detected the presence of CBZ in surface and groundwater at levels up to 18 ng L^−1^. Having this in consideration, water reclamation from the effluents of MWTPs should encompass effective methods for the removal of CECs. In this context, increasing interest has been directed to the application of advanced oxidation processes [3,16,17]. The application of such processes is claimed to accomplish the quality criteria for wastewater reuse [18], through the production of reactive oxidative species improving the degradation and removal of CECs from municipal wastewaters. One of the most applied process is ozonation, which involves both direct oxidation by the molecular ozone and indirect oxidation by the generated hydroxyl radicals [19,20]. In some countries, such as Switzerland, this technology is already implemented at large scale as a tertiary treatment to remove PPCPs [21,22]. Despite this, the ozonation process has some disadvantages, as it can lead to the production of refractory by-products with higher toxicity than parent compounds. For example, Gomes et al. [23] reported that ozonation of a mixture of parabens resulted in a treated solution with higher ecotoxicity to aquatic species than the parent solution, due to the formation of toxic by-products. Therefore, it is important to evaluate the impact of ozone over PPCPs abatement, but also on the ecotoxicity of the treated solutions for a wide range of representative species.

The extent of oxidation of the ozonation process depends on several factors, including the type of contaminants, the water matrix composition, the ozone reaction time and ozone dose [20,24,25]. These aspects must be considered in the development and optimization of the ozonation process for wastewater treatment, as they affect the removal efficiency. Herein, we addressed the effect of the water matrix composition and the ozone reaction duration. The novelty of the present work concerns the assessment of the effect of the water matrix (ultrapure water vs municipal wastewater) on the degradation of five PPCPs (methylparaben (MP), propylparaben (PP), PCT, SMX and CBZ) following ozonation, as well as the effect of complexifying the contaminants’ mixture in the water matrix by adding cumulatively up to five PPCPs. Furthermore, the effect of the ozone reaction time on the toxicity of the treated water (treatments applied to spiked municipal wastewater to avoid unrealistic exposure scenarios as provided by synthetic effluents prepared in ultrapure water) over a range of model species (the microalgae *Raphidocelis subcapitata*, the macrophyte *Lemna minor*, the crustacean *Daphnia magna* and the watercress *Lepidium sativum*) was also assessed, ultimately aiming to determine the optimal end reaction point. Specific objectives were: (i) to assess the degradation kinetics (pseudo-first order kinetic rate constants) of PPCPs, individually and in mixture, in ultrapure water; (ii) to assess the degradation kinetics (pseudo-first order kinetic rate constants) of PPCPs, individually and in mixture, in a secondary wastewater from a MWTP; and (iii) to assess the ecotoxicological impact of the ozonation reaction time (0–45 min) in a municipal wastewater added to the five PPCPs. The secondary municipal wastewater was used comparatively with ultrapure water to reflect the realistic increase in matrix complexity faced in real scenarios.

## 2. Materials and Methods

### 2.1. Test Chemicals and Water Matrices

The selected pharmaceutical and personal care products were obtained from Sigma-Aldrich (≥99% purity). The main characteristics of these compounds (chemical structure, solubility and hydrophobicity) can be retrieved from Gomes et al. [26]. These contaminants were added to ultrapure water or to a municipal water sample to generate the treatment matrices used in the study.

The municipal wastewater (MW) sample was used as matrix to represent test conditions close to a real scenario. It corresponded to a secondary effluent collected from a MWTP where the secondary treatment consists in activated sludge. The properties of the collected effluent were as follows: chemical oxygen demand (COD) = 65 mg O_2_ L^−1^; biochemical oxygen demand (BOD_5_) = 22 mg O_2_ L^−1^; total nitrogen = 40 mg L^−1^; pH 7; total (suspended plus dissolved) solids = 15 mg L^−1^. The addition of the five PPCPs at the concentration of 1 mg L^−1^ to the municipal wastewater increased the COD values to 95 mg O_2_ L^−1^.

### 2.2. Assessment of Ozonation Efficacy

#### 2.2.1. Ozonation Conditions

The ozonation experiments were performed at constant temperature (25 ± 1 °C) in a 2 L glass reactor with continuous stirring at 700 rpm [23]. The reaction time was established in accordance to the contaminant/mixture used, in order to reach a complete degradation of the added PPCPs. The inlet and outlet ozone gas concentrations were followed during the reactions by two ozone gas analyzers (BMT 963 vent and BTM 964 vent), which allowed the determination of transferred ozone dose (TOD) in accordance with Equation (1):

(1) $$\mathrm{TOD}=\int_{0}^{t}\frac{Q_{\mathrm{Gas}}}{V_{\mathrm{liquid}}}\times \left({\left[O_{3}\right]}^{\mathrm{in}}-{\left[O_{3}\right]}^{\mathrm{out}}]\right)\times \mathrm{dt}$$

The [O_3_]^in^ and [O_3_]^out^ represent, respectively, the inlet and outlet ozone concentrations, in mg L^−1^, where V_liquid_ is the volume of solution to be treated and Q_Gas_ is the inlet gas flow rate (12 L h^−1^). Ozone was produced from pure oxygen (99.9%) using an ozone generator (802N, BMT). The residual ozone gas which leaves the reactor was trapped by an aqueous solution with 2% of potassium iodide.

#### 2.2.2. Determination of Degradation Kinetics

The degradation of PPCPs was assessed in both water matrices: ultrapure water and the MW. The pH was kept unchanged. Different synthetic effluents were prepared to represent increased complexity by increasing the number of PPCPs in the solution from 2 to 5: Mix2 included MP and PP; Mix3 included MP, PP and PCT; Mix4 included MP, PP, PCT and SMX; and Mix5 included MP, PP, PCT, SMX and CBZ. Each PPCP was added at a concentration of 1 mg L^−1^. In each matrix the degradation was assessed for single PPCPs and for the abovementioned mixtures. The volume used for the experiments with spiked ultrapure water and spiked MW was 2 L. The experiments were performed in duplicate and the standard deviation was lower than 5%.

#### 2.2.3. Ecotoxicity Assessment

The ecotoxicity was assessed using the aquatic model species *R. subcapitata*, *L. minor*, *D. magna*, and the terrestrial plant *L. sativum*. These species are representative of groups of organisms possibly affected by the discharge of treated municipal wastewater in aquatic systems or following its use for irrigation in the case of watercress. A brief overview of the experimental procedure of each test is depicted in Figure S1 (Supplementary Material).

The ecotoxicity assessment was performed for the MW added the five PPCPs (MP + PP+ PCT + SMX + CBZ) at 1 mg L^−1^ each, submitted to ozonation for different periods of time (0, 2, 4, 8, 15, 20, 25, 30 and 45 min of reaction). This time range was selected following evidence from the kinetic studies, ensuring the complete degradation of the five PPCPs added to the effluent (cf. Section 3.2.1). The pH was kept unchanged. Samples for this assessment (100 mL each) were taken from the glass reactor after the corresponding reaction time and were frozen until being tested. Furthermore, the raw MW (no PPCPs added) was also tested for comparison.

Given that the effluent might not contain the nutrients required for growth of the test species, the samples were spiked with nutrients previously to the ecotoxicological tests to reach the same nutrient levels as supplied in their standard test medium. This procedure assured that any observed ecotoxicological effect was due to the presence of contaminants in the sample rather than to nutrient scarcity. The nutrient spiking caused a slight dilution of the sample, as identified in Table S1 (Supplementary Material). Considering that the test medium for *Lepidium sativum* germination and growth is distilled water], no nutrient spiking was performed for this ecotoxicological test (Table S1-Supplementary Material).

The growth inhibition assay with the microalga *R. subcapitata* was performed following the OECD guideline 201 [27], with the modifications detailed by Gomes et al. [23]. Four days before starting the assays, an inoculum was harvested from the bulk microalgae culture (batch cultures in Woods Hole MBL medium at 20 ± 1 °C, under a 16 h light: 8 h dark photoperiod cycle) and incubated at 23 ± 1 °C under continuous illumination (approximately 7000 lux). This inoculum was used to start the test, with a cell density of 1.0 × 10^4^ cells mL^−1^ per replicate. Owing to the nutrient spiking samples were tested at 98.2% strength (Table S1). The control consisted of ultrapure water, nutrient spiking, and microalgae. Each sample was tested in triplicate. Assays were carried out in 24-wells microplates. Microalgae were exposed to the test samples during 96 h at 23 ± 1 °C and under continuous light supply (approximately 7900 lux). After this period, their growth was assessed based on the absorbance of each sample at 440 nm (spectrophotometer Shimadzu UV-1800, Kyoto, Japan). The absorbances were converted to algal density using a previously defined calibration curve, which were further used to determine the biomass yield (cells mL^−1^).

The growth inhibition assay with *L. minor* was performed following the OECD guideline 221 [28], with the modifications described by Gomes et al. [23]. Laboratory cultures were maintained in Steinberg medium at 20 ± 1 °C under a 16 h light: 8 h dark photoperiod cycle). Assays started with three colonies with three fronds each per replicate. Each sample was tested in triplicate, except control, which was tested in sextuplicate. Due to nutrient spiking, the tested samples were tested at 93.5% strength (Table S1). The control consisted of ultrapure water, nutrient spiking, and *L. minor* colonies. Assays were carried out in 6-wells microplates. Macrophytes were exposed to the test samples during 7 days at 23 ± 1 °C under continuous illumination (approximately 7900 lux). After this period, the number of fronds per replicate was determined, and their dry weight was determined after drying at 60 °C. The estimation of the dry weight of macrophytes at the beginning of the tests was performed by applying the same dry-weighing procedure to seven groups of three colonies with three fronds, each sampled from the same batch culture used to feed the test. The number of fronds and the dry weight were used to determine both yield inhibition and growth rate inhibition, following the OECD guideline 221.

The immobilization assay with *D. magna* was carried out following the OECD guideline 202 [29] with the modifications detailed by Gomes et al. [23]. Laboratory cultures were maintained in ASTM hard water [30], supplied with an organic additive extracted from *Ascophyllum nodosum*, at 20 ± 1 °C under a 16 h light: 8 h dark photoperiod cycle (light intensity about 300 lux) and fed every other day with *R. subcapitata* at 3.0 × 10^5^ cells mL^−1^. Neonates (<24 h old) from the 3rd to 5th brood were used in the assays (five individuals per replicate). Owing to nutrient spiking samples were tested at 92.0% strength (Table S1). A control treatment, consisting of ultrapure water, nutrient spiking and daphnids was also performed. Each treatment was tested in quadruplicate and the assays were carried out in glass test tubes. Daphnids were exposed to the test samples during 48 h, in the absence of food, under the same temperature and photoperiod conditions as described for the cultures. After this period, daphnids immobilization was assessed and the percentage of immobilization was determined.

The germination assay with *L. sativum* was performed to evaluate seed germination and radicle growth, following Gomes et al. [23]. Seeds were obtained from a local supplier and kept in a dry location until use. Seeds were sown in a Petri dish containing a paper filter dipped in 5 mL of sample, using 10 seeds per replicate. A control treatment was also carried out, consisting of ultrapure water. Each sample was tested in duplicate. Seeds were exposed to the test samples during 48 h, at 27 ± 1 °C in the dark. After this period, the number of germinated seeds in each sample (Nt) and in the controls (Nc) were recorded. Seeds were considered germinated if the radicle was visible or, at least, there were clear signs of germination, namely a crack in the seed coat. If the radicle was visible, its length was measured in each sample (Lt) and in the controls (Lc) using a digital caliper. The data were used to determine the percent inhibition of seed germination (G) following ISO [31]:

G (%) = (Nc − Nt)/Nc × 100(2)

The inhibition of radicle growth relative to the control was expressed as the percent phytotoxicity (P), following Sahu et al. [32]:

P (%) = (Lc − Lt)/Lc × 100(3)

The percentage of relative seed germination (RSG), relative radicle growth (RRG), and the germination index (GI) were also determined, according to Trautmann and Krasny [33]:

RSG (%) = Nt/Nc × 100(4)

RRG (%) = Lt/Lc × 100(5)

GI (%) = (RSG (%) × RRG (%))/100(6)

### 2.3. Analytical Methods

To follow the PPCPs concentration along the ozonation experiments, a high-performance liquid chromatography (HPLC) was used (Beckman-System Gold). The SMX was detected at λ = 280 nm whereas both parabens, CBZ and PCT were detected at λ = 255 nm. The mobile phase consisted in a mixture of 50:50 acidic water (0.1% orthophosphoric acid): methanol with the injection volume of 100 μL. A C18 (SiliaChrom) chromatography column was used with the oven at 40 °C. All samples were filtered with a syringe cellulose acetate filter (pore size 0.45 μm) before being analyzed.

For the experiments with municipal wastewater as matrix the COD was used as an indicator of the organic matter content. This parameter was determined according to the standard method 5220D [34]. The COD analysis was run in duplicate and the absorbance of the solution was determined with a spectrophotometer (Lovibond MD 600) at λ = 430 nm. The calibration curve was prepared using a potassium hydrogen phthalate for the COD range 0–100 mg O_2_ L^−1^.

## 3. Results and Discussion

### 3.1. Ozonation of PPCPs, Single and in Mixture, in Ultrapure Water

Ozonation is one of the most used advanced oxidation processes for disinfection and decontamination, due to the high oxidative potential of molecular ozone [3,16]. Moreover, at neutral pH, ozone can partially decompose to produce hydroxyl radicals, which increase the removal efficiency of recalcitrant compounds [19]. The ozone action depends highly on the compounds to treat owing to its electrophilic character, which renders it preference by saturated bonds, amine groups and aromatic rings [20]. The kinetic rates for the degradation of single compounds were evaluated following previous studies [35,36]. Moreover, in the case of oxidation through ozone action, these constants were estimated both in function of TOD and time. This is relevant since the ozone action is more affected by TOD than time given that ozone consumption is the primary operational parameter in such reaction [37].

The pseudo-first order kinetic rates in function of time or TOD were very similar for all compounds except CBZ, which showed higher kinetic rates (Table 1). The CBZ molecule has a tricyclic aromatic structure and amino groups which makes it very susceptible to the ozone action [38,39], thus explaining the higher kinetic rates. The lower value of k’_1,TOD_ for PCT means that a higher amount of ozone was used to accomplish its total removal which will influence the pseudo-first order kinetic rate obtained in function of time. Therefore, for the ozonation reaction, these kinetic rate constants based only on the concentration of microcontaminants (pseudo-first order) should be compared also in terms of the transferred ozone dose, since time can mask the results. Considering a future scale-up approach, this can denote a deviation in the operational and installed conditions, which might represent additional costs.

Gomes et al. [26] determined the pseudo-first order constant for the same compounds using ozone at pH 3, where the main oxidative species is the molecular ozone, and they obtained similar values, except for the parabens. Indeed, the values of k’_1_ were half than those obtained in the present study for both parabens, which can be related to the low selectivity of molecular ozone for these compounds. Tay et al. [40] determined the second order kinetic constants for ozone (kO_3_, M^−1^ s^−1^) at pH 6 and the values obtained were 2.5 × 10^5^ and 4.1 × 10^5^ M^−1^ s^−1^ for MP and PP, respectively. The second order rate constants for k_O3_ regarding these two compounds were lower comparing to PCT and SMX, respectively, 2.57 × 10^6^ M^−1^ s^−1^ [36] and 2.60 × 10^6^ M^−1^ s^−1^ [41]. Moreover, Huber e al. [41] also determined the kO_3_ for CBZ but the value was lower (3 × 10^5^ M^−1^ s^−1^), similarly to the parabens. These values do not agree with the kinetic rate constants determined in the present study, which can be explained by the water matrix considered. In the present study, ultrapure water was used with only CBZ added, whereas Huber et al. [41] used real wastewater. This will be further discussed when addressing the results of the degradation kinetics in MW. Besides this, Giri et al. [42] determined a pseudo-first order kinetic rate of about 1.07 min^−1^ for the ozonation of 1 mg L^−1^ of CBZ, which is similar to the obtained in the present study (Table 1).

**Figure 1:**
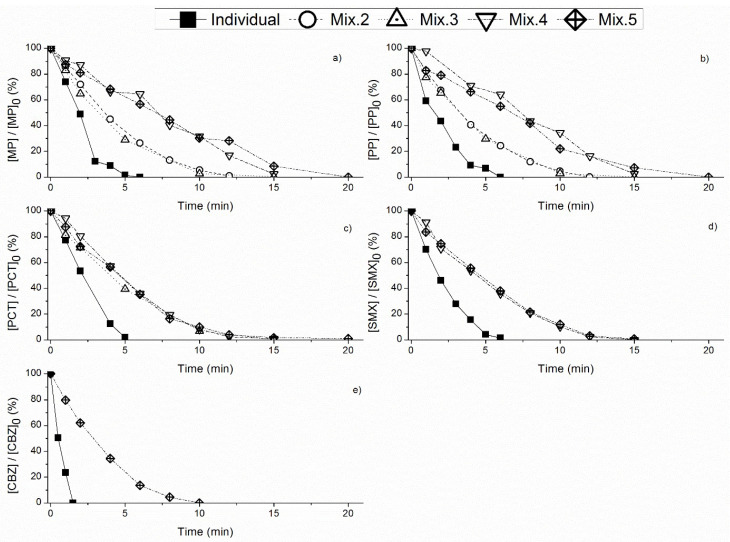
Normalized concentration of: (**a**) MP; (**b**) PP; (**c**) PCT; (**d**) SMX; (**e**) CBZ, during ozonation of individual PPCPs and the corresponding mixtures (Mix2, Mix3, Mix4 and Mix5), throughout the reaction time, using ultrapure water as matrix at pH 5.5 and initial concentration of each PPCP 1 mg L^−1^. Mix2 included MP and PP; Mix3 included MP, PP and PCT; Mix4 included MP, PP, PCT and SMX; and Mix5 included MP, PP, PCT, SMX and CBZ.

The removal of each PPCP, individually and in mixture, throughout the reaction time, is depicted in Figure 1 and the corresponding pseudo-first order kinetic rate constants are presented in Table 2.

Comparing to the individual compounds, the addition of contaminants decreased the kinetic rates of each contaminant in mixture, for all mixtures (Table 2). Regarding the degradation of the parabens, increasing the number of contaminants from 2–3 to 4–5 caused a pronounced decrease in their degradation (Figure 1, Table 2). However, in the case of PCT, such variation was not observed, and the degradation rates were similar for the mixtures with 3, 4 and 5 contaminants (Figure 1, Table 2). The pseudo-first order kinetic rates were very similar for PCT and SMX concerning Mix3, Mix4 and Mix5 (Table 2). The second order kinetic rate constants for kO_3_ are very similar for both contaminants: 2.57 × 10^6^ M^−1^ s^−1^ [36] and 2.60 × 10^6^ M^−1^ s^−1^ [41], respectively. This suggests that the pseudo-first order kinetic rates determined in the present study followed the same trend. In Mix5 the highest decrease in terms of the degradation profile, as well as in the pseudo-first order kinetic rates, was observed for MP, PP and CBZ (Figure 1, Table 2). This agrees with the previous conclusion regarding SMX and PCT, since MP, PP and CBZ have similar second order constants [40,41]. However, even in mixture CBZ continues to be the most quickly degraded compound due to its higher electrophilicity.

The TOD values followed the same pattern as presented in Figure 1. Increasing the number of PPCPs in mixture promoted an increase in TOD values. This means that more ozone was consumed to achieve the complete degradation of all contaminants, which agrees with the literature [21,26,37]. For example, comparing Mix4 and Mix5, the time to achieve the complete removal of all contaminants was 20 min in both mixtures, but the TOD value was 20.8 mg O_3_ L^−1^ for Mix4, and 25.1 mg O_3_ L^−1^ for Mix5. This suggests that increasing the number of PPCPs in a mixture will require more ozone to achieve their complete degradation.

### 3.2. Ozonation of PPCPs in Municipal Wastewater

#### 3.2.1. PPCPs Removal

**Figure 2:**
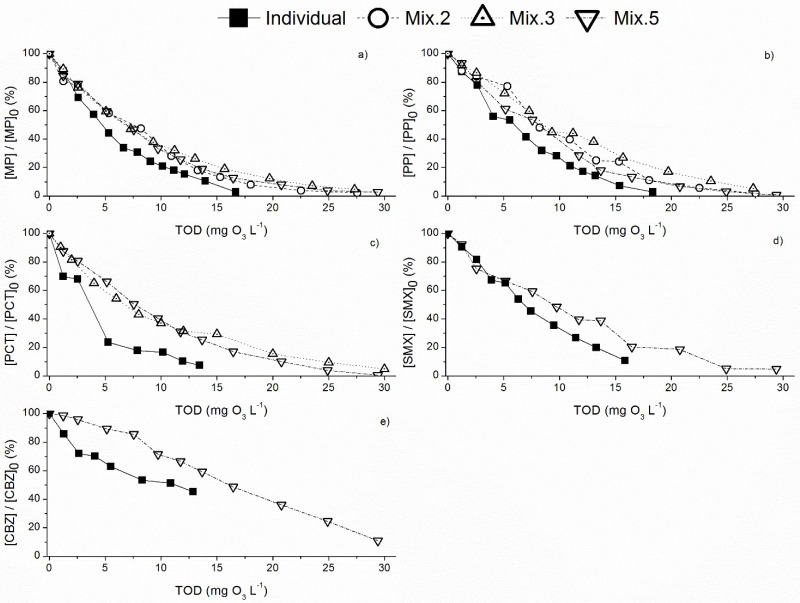
Normalized concentration of: (**a**) MP; (**b**) PP; (**c**) PCT; (**d**) SMX; (**e**) CBZ, during ozonation of individual PPCPs and the corresponding mixtures (Mix2, Mix3 and Mix5), throughout the TOD values, using a municipal wastewater as matrix at pH 7 and initial concentration of each PPCP 1 mg L^−1^. Mix2 included MP and PP; Mix3 included MP, PP and PCT; and Mix5 included MP, PP, PCT, SMX and CBZ.

The municipal wastewater presents a wide range of organic and inorganic compounds due to the inefficacy of conventional treatments [11,12,16]. The presence of such compounds can influence the efficiency of the ozonation process [43]. The presence of organic matter leads to a reduction in the ozone activity due to trapping of the ozone molecules, reducing the decomposition into hydroxyl radical species. Moreover, inorganic species such as HCO_3_^−^, SO_4_^2−^ and Cl^−^ can also contribute to reduce the ozone action [44]. Some of these species act as radical scavengers during ozonation, consuming the produced hydroxyl radical and the molecular ozone, which decreases the contaminants’ degradation rate [45]. Hence, the degradation profile by ozonation was assessed for each PPCP, individually and in mixture (Mix2, Mix3 and Mix5), using municipal wastewater (MW) as matrix. As in terms of PPCPs degradation profile the difference between Mix4 and Mix5 was small, it was just presented for the more complex mixture Mix5. The removal of each PPCP throughout the reaction time is depicted in Figure S2 (Supplementary Material), and their removal in function of the ozone consumption is depicted in Figure 2.

Contrary to the results obtained using ultrapure water as matrix (Figure 1), the degradation profiles using wastewater as matrix did not undergo noticeable differences with increasing the number of compounds in the mixture (Figure 2), except if comparing to PCT when used alone (Figure 2c). This suggests that the degradation kinetics of PPCPs seems to stabilize when a complex matrix is used with different inorganic and organic compounds, likely including other unmonitored contaminants. The pseudo-first order kinetic rate constants (Table 3) support these results, indicating similar values for the different mixtures considered. The degradation rate of each individual PPCP suffered a significant decrease when the MW was used as matrix comparing to the ultrapure water. This is related to the presence of organic and inorganic species that trap the ozone action and its decomposition into radical oxidative species. In fact, TOD values obtained for all the reactions with the MW as matrix were higher than for reactions with ultrapure water. However, this difference decreased as the mixture complexity increased. For instance, regarding Mix3, the TOD values using MW or ultrapure water as matrix were 13.4 mg O_3_ L^−1^ and 6.6 mg O_3_ L^−1^, respectively, i.e., comprising a two-fold difference; for Mix5 the TOD values were 29.4 and 25.1 mg O_3_ L^−1^, respectively, for MW and ultrapure water. Considering the pseudo-first order kinetic rates for the MW and ultrapure water, the constants for MP, PP and PCT were also about twice as high in the MW than in ultrapure water (Table 2 and Table 3). This clearly indicates that the constants obtained here are very dependent on the TOD values, since molecular ozone will be the main responsible by the PPCPs oxidation. The degradation performance of CBZ, individually, presented the most notable reduction when comparing both matrices: in MW about 50% of removal was achieved after 10 min of reaction (Figure 2e), whereas in ultrapure water a total degradation was achieved in the same time (Figure 1e). The presence of organic and inorganic species in MW inhibits the ozone action over electrophilic species like CBZ, which is not so pronounced in the case of MP and PP. Moreover, according to the second order kinetic constants (k_O3_) for MP, PP and CBZ, 2.5 × 10^5^ M^−1^ s^−1^, 4.1 × 10^5^ M^−1^ s^−1^ [40] and 3.0 × 10^5^ M^−1^ s^−1^ [41], respectively, similar degradation profiles were expected, which was not observed (Figure 2a,b,e). Moreover, the second order kinetic constants for hydroxyl radicals (k_OH_) are from 4.9 × 10^9^ M^−1^ s^−1^ (for PCT) [36] and 8.8 × 10^9^ M^−1^ s^−1^ (for CBZ) [16]. According to the pseudo-first order kinetic rate constant for individual PPCPs and mixtures (Table 3), it is possible to see that the degradation does not follow the same order, since CBZ presents the smallest values, which does not agree with the second order k_OH_. These results obtained with MW suggest that the presence of organic matter decreases significantly the efficiency of the ozonation process over the degradation of highly electrophilic PPCPs.

The pseudo-first order kinetic rates also support this conclusion. In fact, the k’_5_ for CBZ (Mix5) was 0.05 min^−1^ in MW (Table 3) and was 0.30 min^−1^ in ultrapure water (Table 2). On the other hand, the k’_5_ for MP and PP (in Mix5) was similar for both MW and ultrapure water (Table 2 and Table 3), which suggests that the presence of organic and inorganic species has a minor effect on the degradation of poorly electrophilic compounds, but a pronounced effect on the degradation of highly electrophilic compounds. The degradation of MP and PP was more affected by the presence of other electrophilic PPCPs. Regarding PCT and SMX, the values of pseudo-first order kinetic rates in both water matrices were slightly different, with the values in MW being around the double of the values in ultrapure water.

Another relevant feature is that adding more PPCPs to the mixture in MW from two until five did not change noticeably the respective pseudo-first order constant values. Therefore, for secondary effluent matrices, it can be considered that the addition of PPCPs did not affect significantly the pseudo-first order kinetic rates of individual PPCPs. This is an important indication for the scale-up of these treatment technologies. In fact, the kinetic constants obtained for the MW matrix can be considered as an initial approach to predict the treatment operations and requirements to achieve the removal of PPCPs from wastewater.

#### 3.2.2. COD Removal

**Figure 3:**
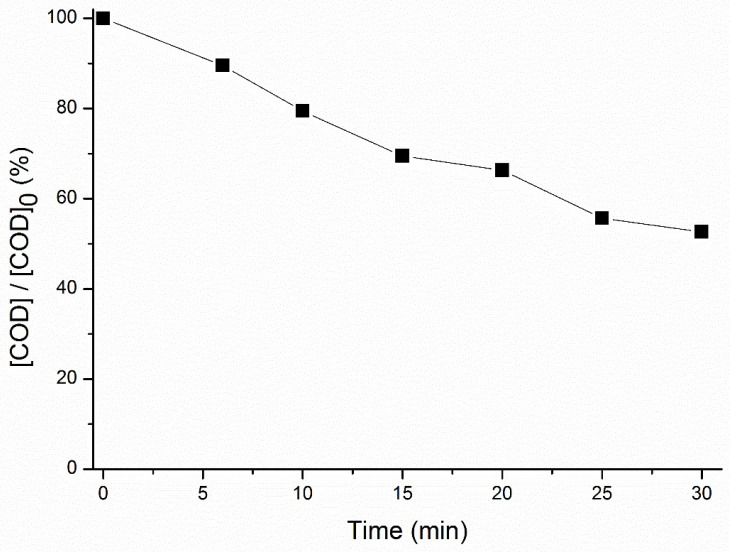
COD removal for the MW (pH 7) spiked with five PPCPs (MP, PP, PCT, SMX and CBZ), at 1 mg L^−1^ each, during ozonation.

Besides the degradation of each PPCP, it is important to evaluate the organic matter load during the application of advanced oxidation processes, which was addressed herein through the measurement of COD. This allows an understanding on whether PPCPs degradation is achieved by the partial oxidation or mineralization pathway: a low COD removal along the reaction time can be related to the formation of refractory by-products which suggests partial oxidation instead of mineralization [46]. The COD was followed along the reaction for the MW spiked with the mixture of five PPCPs (Figure 3).

The COD removal for the time considered (30 min) was of about 53%, which represents a value of 50.4 mg O_2_ L^−1^. This value was even lower than the initial COD value in the raw MW (without the PPCPs addition, 65 mg O_2_ L^−1^), which suggests that some organic matter of the MW was degraded in parallel to the added PPCPs. Moreover, under these conditions, CBZ and SMX did not achieve a complete degradation, also suggesting the consumption of ozone for degradation of other organic species besides the added PPCPs. Moreover, the COD removal attenuated from the 15 min onwards (Figure 3), which indicates that the produced by-products were more difficult to remove by the ozonation process. Hence, besides the COD removal, it is also important to evaluate the toxicity of the ozone-treated solutions as an ultimate confirmation regarding the efficiency of ozonation in allowing the discharge of environmentally safe effluents.

#### 3.2.3. Ecotoxicity Evaluation

**Figure 4:**
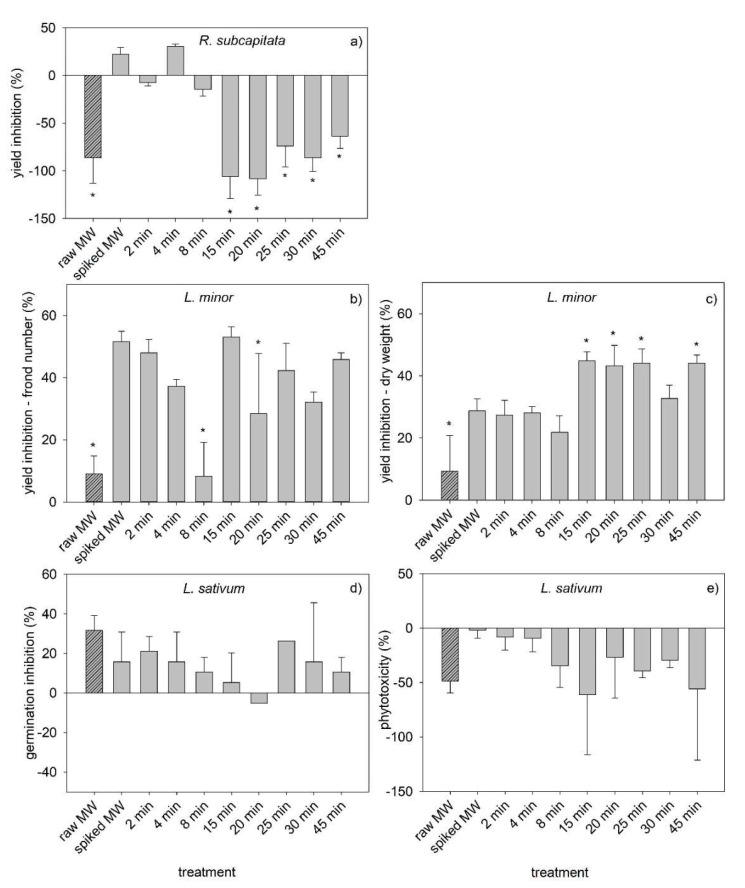
Toxicity response of several model species exposed to the raw municipal wastewater (raw MW, diagonal lines), and to the MW added five PPCPs (MP, PP, PCT, SMX and CBZ at 1 mg L^−1^ each) before ozonation (spiked MW) and after different periods of ozonation. (**a**) Yield inhibition of *R. subcapitata* after 96 h of exposure; (**b**) yield inhibition based on the frond number of *L. minor* after 7 d of exposure; (**c**) yield inhibition based on the dry weight of *L. minor* after 7 d of exposure; (**d**) germination inhibition of *L. sativum* after 48 h d of exposure; and (**e**) phytotoxicity of *L. sativum* after 48 h of exposure. Bars represent the mean and the error bars represent the standard deviation. Asterisks represents significant differences relative to the untreated spiked MW.

Ecotoxicity of the municipal wastewater was analyzed under different conditions: original untreated MW (raw MW); MW spiked with the mixture of five PPCPs before the ozonation treatment (spiked MW) and at different time points during the ozonation reaction (representing worst-case scenarios in terms of treatment requirements). The results of the ecotoxicity evaluation are presented in Figure 4 and Figure S3 (Supplementary Material).

Toxicity to the microalga *R. subcapitata* (Figure 4a) was low, with only two samples exhibiting yield inhibition (untreated sample and t = 4 min). For the remaining samples, the yield inhibition values were negative, meaning that there was increased yield rather than inhibition compared to the control. This is concordant with the results of a previous review highlighting that ozonated wastewater samples commonly elicited no adverse effects on the growth of this microalga species [47].

Microalgae yield inhibition showed a general trend to decrease as the duration of the ozonation increased. The exception was at t = 4 min, where an increased yield inhibition (32% yield inhibition) was noticed compared to the spiked MW (22% yield inhibition). The trend for a decreased toxicity of the spiked wastewater to *R. subcapitata* during ozonation was also observed in a previous study with a secondary effluent added the pharmaceutical ofloxacin, despite some fluctuations [24]. Interestingly, ozonation was efficient in reducing the toxicity of the spiked MW for all ozonation periods, except for t = 4 min, despite these differences were statistically confirmed only for t ≥ 15 min. Moreover, ozonation was able to further reduce toxicity compared to the raw MW, as the yield inhibition observed after 15, 20 and 30 min (−106%, −108% and −87%) was equal or lower than the yield inhibition of the raw MW.

Yield inhibition for ozonation periods ≥ 15 min was markedly lower than for ozonation periods ≤ 8 min. This might be due to the degradation of toxic PPCPs or other organic compounds in the MW, and/or to the formation of degradation by-products promoting microalgae growth. Among the tested PPCPs, only SMX shows EC_50_ values (after a 72 h or 96 h exposure period) below 10 mg L^−1^ (see summary of the EC_50_ values for each species regarding exposure to each PPCP in Jesus et al. [48]. Indeed, EC_50_ values as low as 0.146 mg L^−1^ [49] were found. Since SMX is degraded over time, and considering the pronounced decrease in its concentration between 8 min and 15 min (in this period, SMX concentration decreased from about 50% to about 20% of its initial concentration, Figure 2d), it is likely that SMX degradation contributed substantially for the microalgae growth observed in the ozonation period 15–45 min. On the other hand, the hypothesis of the formation of degradation by-products is supported by the observed attenuation of COD removal from 15 min onwards (Figure 3). The toxicity observed at t = 4 min might be due to the formation of degradation by-products, as well. A vast number of by-products has been reported to form following ozonation of the five PPCPs added to the effluent that can be more toxic than the parent compounds [48]; this can be exacerbated by the likely presence of other organic compounds in the effluent and the consequent formation of other by-products.

A previous study assessed the toxicity of the same mixture (the same five PPCPs at 1 mg L^−1^ each), but prepared in ultrapure water, before and after a 60 min ozonation reaction [48]. A comparison of the response of each species to these PPCPs using these water matrices (ultrapure water and MW) is provided in Table S2, allowing a comparison of the water matrix effect on the ecotoxicological response of the tested species. Results seem to confirm that MW was more favorable to microalgae growth (also, raw MW per se promoted microalgae growth). This might be due to the presence of low metal concentrations, which can stimulate the growth and metabolism of microalgae, a phenomenon known as hormesis, or to the presence of nutrients such as nitrates and phosphorous [50]. However, concerning the ozone-treated samples, the different ozonation periods (45 min in the present study; 60 min in Jesus et al. [48]) might also contribute to explain the lower toxicity observed in the present study. Indeed, in a previous study [23], it was reported that increased time of ozonation of a mixture of five parabens tended to decrease the toxicity to this microalga species over time (most notably in the period 80–120 min). Despite the ozonation period in the present study did not reach 80–120 min, we cannot exclude the possibility of changes in the toxicity owing to the ozone reaction duration. In addition, the reduced toxicity of the ozonated MW can be also due to potential antagonistic interactions among the added and pre-existing compounds in the effluent and their degradation by-products [51]. One may argue that differences in the ecotoxicological response of the test species might be attributed to the pH value. However, the pH of the test samples after nutrient spiking was very similar (close to the neutral value) both for the samples in ultrapure water (previous study, [48]) and in MW, which is due to the high buffering capacity of the compounds added to the test media (nutrient spiking). Thus, pH effect would not have been relevant to explain the different ecotoxicological responses in both water matrices.

Toxicity to the macrophyte was determined as yield inhibition, both as a function of the number of fronds (Figure 4b) and dry weight (Figure 4c). Despite the differences in the responses, there are similar trends. In both cases, there was inhibition rather than growth stimulation, oppositely to the microalgae response. Indeed, *L. minor* was negatively affected by exposure to untreated and ozone-treated effluents in several studies e.g., [52], as reviewed by Völker et al. [47]. Moreover, in both cases, increased toxicity was observed after addition of the mixture of PPCPs, comparing to the raw MW. Additionally, there is a distinction in the toxicity observed for ozonation durations ≤8 min and ≥15 min, more pronouncedly when considering the yield inhibition as a function of dry weight (Figure 4c). Unlike the effect observed for the microalgae, toxicity is higher in the period 15–45 min rather than in the period 2–8 min. Moreover, for both endpoints, there is a trend for decreasing toxicity with increasing ozonation duration during the first 8 min of reaction, similarly to the observed for the microalgae, probably due to SMX. As for the microalgae, SMX is toxic for *L. minor*, with a 7d-EC_50_ value of 1.48 mg L^−1^ [53] regarding yield inhibition in function of frond number. Thus, decreasing SMX concentration is likely the main explanation for the decreasing toxicity in that period.

The maximum value of yield inhibition was observed in the sample ozonated during 15 min, reaching a value of 53% based on frond number and 34% based on dry weight. This might be related to the formation of toxic by-products, as supported by Figure 3, both deriving from the added PPCPs or other organic compounds in the MW. The lack of studies addressing the toxicity of ozonation by-products to *L. minor* prevents further discussion of the likely causes of toxicity. Take the example of p-benzoquinone, which is an ozonation by-product of several PPCPs added to the effluent: parabens, PCT and SMX [48]. This compound could be considered a potential cause for the observed toxicity to the macrophyte but the absence of ecotoxicological studies to macrophytes prevents to appraise whether p-benzoquinone is likely responsible for the observed toxicity.

Ozonation was efficient in reducing the toxicity of the spiked effluent in the period 2–8 min regarding both endpoints, despite statistically significant differences were observed only for t = 8 min regarding the yield inhibition expressed in function of the frond number (Figure 4b). Moreover, ozonation reduced toxicity compared to the raw MW for this single exposure period (t = 8 min) regarding this endpoint. Note that for the microalgae, this was observed for three time points.

Comparing the effects of the water matrix on the toxicity to *L. minor*, only minor effects were found (Table S2), with a trend for increased toxicity in the MW rather than in ultrapure water, suggesting that potentially nutritional benefits of MW compared to ultrapure water were not enough to overcome the toxicity of the contaminants present (notice the 15% yield inhibition caused by the raw MW in Figure 4c).

Regarding the effects on the watercress, the results for the endpoints germination inhibition (Figure 4d) and phytotoxicity (Figure 4e) are opposite: the first showed inhibition whereas the later showed stimulation, with a single exception. These results mean that exposure to the samples inhibited germination of seeds but increased the length of the radicles of those seeds that were successful in germinating. Despite no significant differences relative to the control were observed for any of these endpoints, data showed a trend for decreased toxicity over time until t = 15 min (phytotoxicity) or t = 20 min (germination inhibition). This trend for reduced toxicity over time, followed by an increase and then a gradual decrease, was observed for both endpoints, and is concordant with the response of the two other primary producers (the microalgae and the macrophyte). Very few data exist on the ecotoxicological assessment of chemicals to *L. sativum*, which makes any further discussion on the main responsible chemicals for such toxicity speculative.

Interestingly, the addition of the PPCPs to the effluent tended to decrease germination inhibition, reflecting low toxicity potential of the added PPCPs. The same mixture added to ultrapure water caused also a minor germination inhibition (about 10%, [48]). Note, however, that the phytotoxicity showed an increase due to addition of the PPCPs, which is concordant with the response of the other primary producers. The main responsible for this increase might be SMX, as this PPCP was reported to cause a phytotoxicity of about 55% to the watercress [48], as well as considering that SMX seems to be a major actor in increasing toxicity of the spiked MW compared to the raw MW for the microalgae and the macrophyte. Indeed, being primary producers, these species will likely have similar metabolic processes towards the same compound.

Regarding the water matrix effect to *L. sativum* (Table S2), minor effects were observed concerning the germination inhibition endpoint. The picture is significantly different considering phytotoxicity, showing that MW supported a much relevant decrease in phytotoxicity compared to ultrapure water.

Regarding toxicity to *D. magna*, no immobilization was found regardless of the treatment (data not shown), i.e., 0% immobilization was observed for all treatments. This suggests that neither the raw MW nor the spiked MW or the ozone-treated MW were acutely toxic to the daphnids. This was not expected given that 48 h-EC_50_ values as low as 2 mg L^−1^ and 2.99 mg L^−1^ were reported for *D. magna* after exposure to propylparaben and paracetamol, respectively [54,55]. However, in a previous study [48], it was found that the same mixture in ultrapure water was not toxic to this cladoceran, pointing out to antagonistic effects among the added PPCPs [48]. Also, this cladoceran was found to be quite insensitive to exposure to wastewater both before and after ozonation, as reviewed by Völker et al. [47].

Following the above mentioned, the water matrix (ultrapure water vs secondary effluent) had no significant effect on the toxicity of the untreated mixture of PPCPs to *D. magna*. However, after ozone reaction, 12% of immobilization was observed using the matrix ultrapure water [48], whereas no immobilization was observed using the matrix MW (Table S2). Despite 12% being a low value which might not be significant, and considering the different ozonation periods (45 min in the study with ultrapure water, and 60 min in the present study), it supports a trend for ozonation of the MW causing less toxicity to the organisms than ozonation of the same chemicals in ultrapure water.

In summary, the ecotoxicological assessment of the secondary MW added the mixture of PPCPs showed that toxicity varies according to the species, being *D. magna* and *R. subcapitata* the least sensitive, and *L. minor* the most sensitive to the ozonation treatment of the MW. Indeed, *L. minor* yield inhibition reached values above 50% (for t = 15 min regarding the yield inhibition based on frond number). Additionally, toxicity varied widely through the 45 min period, most pronouncedly for the microalgae, with yield inhibition values varying between 30% and −108% (for t = 4 min and 20 min, respectively). Such a wide variability highlights the importance of carefully selecting the most appropriate ozonation duration, when aiming to apply ozonation under real conditions. In this study, there was a trend for decreased toxicity of samples ozonated during 2–8 min for all species, with samples ozonated for 8 min showing lower toxicity than the spiked MW (untreated). This suggests that the optimal end reaction point might be 8 min, as this period elicited good results considering the reduction of toxicity to the tested species. However, having in consideration that the test matrix was a spiked effluent and considering the temporal variability of the effluent composition [56], future studies must be carried out to assess the best duration of the ozone reaction to achieve the best toxicity reduction allied to the lowest TOD, i.e., allying both environmental and economic advantages. In fact, the duration of the ozone reaction should be adjusted to the effluent to be treated, considering the indication provided by periodic ecotoxicological tests. Additionally, this study corroborates that the removal of the parent compounds does not guarantee decreased hazardous potential of effluents to biological species. Indeed, after 45 min of ozonation the added PPCPs were removed (Figure 2) but samples were toxic to *L. minor* (yield inhibition above 40%, Figure 4b,c).

Regarding the water matrix effect, it was observed that the toxicity of the PPCPs was often lower in the MW than in ultrapure water, concerning untreated samples. This suggests that the compounds potentially found in the effluent might have a beneficial effect on the tested species, decreasing toxicity. Despite the slower degradation in the effluent compared to ultrapure water, the ecotoxicological assessment revealed that ozonation might lead to better environmental compatibility using the matrix MW than in ultrapure water, as was observed for the microalgae and for the watercress considering the endpoint phytotoxicity. Hence, toxicity removal might be higher in MW compared to the prediction of studies using ultrapure water as matrix.

For the abovementioned reasons, we highlight the need to further study the environmental impact of ozonation using real effluents rather than ultrapure water, aiming to contribute to a better prediction of the toxicity of ozonated effluents under real operation conditions. Moreover, other species potentially affected by discharge of MWTP effluents must also be considered.

## 4. Conclusions

This study showed that ozonation is, indeed, an efficient process to remove contaminants from the water, highlighting that the time required to achieve a complete removal will increase as the complexity of the water matrix increases, being higher in a MW (about 30 min) than in ultrapure water (≤20 min), under the tested conditions. Despite all PPCPs being almost completely removed from the spiked MW after 45 min of ozonation, this was not the reaction time that showed the best environmental compatibility for the tested species. Indeed, considering all species, an ozone reaction time of 8 min allowed to achieve the highest environmental compatibility, showing reduced toxicity compared to the untreated municipal wastewater. This highlights the extreme importance of considering the toxicity removal besides the removal of specific contaminants.

The temporal variation of the observed effects corroborates the degradation of the added PPCPs and suggests the formation of toxic degradation by-products. For this reason, it is recommended to adjust the ozone reaction time to the wastewater effluent to be treated and further carry out periodic ecotoxicological tests with a wide diversity of species, to ensure that the ozone reaction time is appropriate to achieve the lowest toxicity to the receiving aquatic systems.

In the present study, *Raphidocelis subcapitata* and *Daphnia magna* were the least sensitive species, whereas *Lemna minor* was the most sensitive. The differing response of the species emphasizes the need to include a wide diversity of species in the ecotoxicological assessment of processes for wastewater treatment, focusing not only on aquatic species, but also on plants that can be exposed to the wastewater through irrigation. Moreover, such ecotoxicological assessment should be directed to sublethal endpoints given that species are exposed to the effluents not only for a short period of time but, probably, during their entire life (chronic exposure).

## Figures and Tables

**Table 1:** Pseudo-first order kinetic rate constants of ozone-treated contaminants in ultrapure water (pH 5.5), individually, as a function of time (k’_1_) and as a function of TOD (k’_1,TOD_). The initial concentration of each contaminant (MP, PP, PCT, SMX and CBZ) was 1 mg L^−1^. The corresponding adjusted coefficients of determination (adj R^2^) are also presented.

	MP	PP	PCT	SMX	CBZ
k’_1_ (min^−1^)	0.51	0.50	0.42	0.45	1.56
(adj R^2^)	(0.94)	(0.98)	(0.94)	(0.98)	(0.97)
k’_1,TOD_ (mg O_3_^−1^)	0.40	0.37	0.28	0.38	1.12
(adj R^2^)	(0.94)	(0.99)	(0.91)	(0.97)	(0.97)

**Table 2:** Pseudo-first order kinetic rate constants of ozone-treated contaminants in ultrapure water (pH 5.5), individual and in mixture, as function of time. (k’_1_) refers to the PPCP individually, whereas k’_2_, k’_3_, k’_4_, and k’_5_, refer to Mix2, Mix3, Mix4 and Mix5, respectively (k’_a_, where a is the number of contaminants in the mixture). Mix2 included MP and PP; Mix3 included MP, PP and PCT; Mix4 included MP, PP, PCT and SMX; and Mix5 included MP, PP, PCT, SMX and CBZ at 1 mg L^−1^ each. The corresponding adjusted coefficients of determination (adj R^2^) are also presented.

	MP	PP	PCT	SMX	CBZ
k’_1_ (min^−1^) (adj R^2^)	0.51 (0.98)	0.50 (0.97)	0.42 (0.98)	0.45 (0.95)	1.56 (0.90)
k’_2_ (min^−1^) (adj R^2^)	0.23 (0.98)	0.25 (0.99)			
k’_3_ (min^−1^) (adj R^2^)	0.25 (0.99)	0.25 (0.99)	0.21 (0.99)		
k’_4_ (min^−1^) (adj R^2^)	0.12 (0.94)	0.12 (0.93)	0.20 (0.97)	0.20 (0.98)	
k’_5_ (min^−1^) (adj R^2^)	0.12 (0.97)	0.13 (0.96)	0.20 (0.98)	0.19 (0.98)	0.30 (0.98)

**Table 3:** Pseudo-first order kinetic rate constant of ozone-treated contaminants in MW (pH 7), in mixture, as function of time. (k’_1_) refers to the PPCP individually, whereas k’_2_, k’_3_, and k’_5_, refer to Mix2, Mix3, and Mix5, respectively. Mix2 included MP and PP; Mix3 included MP, PP and PCT; Mix4 included MP, PP, PCT and SMX; and Mix5 included MP, PP, PCT, SMX and CBZ at 1 mg L^−1^ each. The corresponding adjusted coefficients of determination (adj R^2^) are also presented.

	MP	PP	PCT	SMX	CBZ
k’_1_ (min^−1^) (adj R^2^)	0.20 (0.99)	0.19 (0.98)	0.27 (0.96)	0.13 (0.99)	0.08 (0.93)
k’_2_ (min^−1^) (adj R^2^)	0.15 (0.99)	0.12 (0.97)			
k’_3_ (min^−1^) (adj R^2^)	0.11 (0.99)	0.09 (0.99)	0.09 (0.99)		
k’_5_ (min^−1^) (adj R^2^)	0.13 (0.99)	0.13 (0.99)	0.12 (0.99)	0.10 (0.98)	0.05 (0.97)

## Data Availability

All data supporting the results of the present study are reported in the manuscript or as Supplementary Information.

## Supplementary Materials

The following supporting information can be downloaded at: https://www.mdpi.com/article/10.3390/toxics10120765/s1, Figure S1: Laboratorial procedures: (a) immobilization test with *D. magna*; (b) growth inhibition tests with *L. minor*: b1—start of the test, and b2—end of the test; (c) growth inhibition test with the microalgae *R. subcapitata*; (d) germination and growth of *L. sativum*—figure shows the measurement of the length of the root of *L. sativum* with a digital caliper; Figure S2: Normalized concentration of: (a) MP; (b) PP; (c) PCT; (d) SMX; (e) CBZ, during ozonation of individual PPCPs and the corresponding mixtures (Mix2, Mix3 and Mix5), throughout the reaction time, using a municipal wastewater as matrix (pH 7), spiked with 1 mg L^−1^ of each PPCP; Figure S3: Toxicity response of two biological species exposed to the raw municipal wastewater (raw MW, diagonal lines), and to that municipal wastewater added five PPCPs (methylparaben, propylparaben, paracetamol, carbamazepine and sulfamethoxazole) before ozonation (spiked MW) and after different periods of ozonation. (a) growth rate based on the frond number of *L. minor* after 7 d of exposure; (b) growth rate based on the dry weight of *L. minor* after 7 d of exposure; (c) percentage of relative seed germination (RSG) of *L. sativum* after 48 h d of exposure; (d) percentage of relative radicle growth (RRG) of *L. sativum* after 48 h d of exposure; and (e) germination index (GI), expressed in percentage, of *L. sativum* after 48 h d of exposure. Bars represent the mean and the error bars represent the standard deviation. Asterisks represents significant differences relative to the untreated spiked MW. Table S1: Total volume of the nutrient spiking added to each replicate to comply with the standard medium recipe and the corresponding strength of the tested sample (%), for each of the tested species; Table S2: Comparison of the ecotoxicological response of the test species observed after exposure to the mixture of PPCPs (MP, PP, PCT, SMX and CBZ) at 1 mgL^−1^ each, before (untreated sample) and after ozonation (ozone-treated sample) in ultrapure water and MW. The results regarding tests in ultrapure water were obtained from Jesus et al. [48], where the duration of the ozonation reaction was 60 min, whereas the duration of the ozonation reaction in the present study was 45 min. Values highlighted in bold refer to those showing more pronounced differences between the water matrices; in these cases, the values corresponding to the water matrix exhibiting the most favorable response are green shaded.

## References

[1] McNally A., Verdin K., Harrison L., Getirana A., Jacob J., Shukla S., Arsenault K., Peters-Lidard C., Verdin J.P. (2019). Acute water-scarcity monitoring for Africa. Water.

[2] Rosa L., Chiarelli D.D., Rulli M.C., Dell’Angelo J., D’Odorico P. (2020). Global agricultural economic water scarcity. Sci. Adv..

[3] Rizzo L., Gernjak W., Krzeminski P., Malato S., McArdell C.S., Perez J.A.S., Schaar H., Fatta-Kassinos D. (2020). Best available technologies and treatment trains to address current challenges in urban wastewater reuse for irrigation of crops in EU countries. Sci. Total Environ..

[4] Ofori S., Puškáčová A., Růžičková I., Wanner J. (2021). Treated wastewater reuse for irrigation: Pros and cons. Sci. Total Environ..

[5] Krzeminski P., Schwermer C., Wennberg A., Langford K., Vogelsang C. (2017). Occurrence of UV filters, fragrances and organophosphate flame retardants in municipal WWTP effluents and their removal during membrane post-treatment. J. Hazard. Mater..

[6] Saeid S., Kråkström M., Tolvanen P., Kumar N., Eränen K., Mikkola J.-P., Kronberg L., Eklund P., Peurla M., Aho A. (2020). Advanced Oxidation Process for Degradation of Carbamazepine from Aqueous Solution: Influence of Metal Modified Microporous, Mesoporous Catalysts on the Ozonation Process. Catalysts.

[7] Al-Kaf A.G., Naji K.M., Abdullah Q.Y.M., Edrees W.H.A. (2017). Occurrence of paracetamol in aquatic environments and transformation by microorganisms: A review. Chron. Pharm. Sci..

[8] Neamţu M., Bobu M., Kettrup A., Siminiceanu I. (2013). Ozone photolysis of paracetamol in aqueous solution. J. Environ. Sci. Health Part A.

[9] Canosa P., Rodriguez I., Rubi E., Cela R. (2007). Determination of parabens and triclosan in indoor dust using matrix solid-phase dispersion and gas chromatography with tandem mass spectrometry. Anal. Chem..

[10] Lincho J., Martins R.C., Gomes J. (2021). Paraben Compounds—Part I: An Overview of Their Characteristics, Detection, and Impacts. Appl. Sci..

[11] Petrie B., Barden R., Kasprzyk-Hordern B. (2015). A review on emerging contaminants in wastewaters and the environment: Current knowledge, understudied areas and recommendations for future monitoring. Water Res..

[12] Ahmed M.B., Zhou J.L., Ngo H.H., Guo W., Thomaidis N.S., Xu J. (2017). Progress in the biological and chemical treatment technologies for emerging contaminant removal from wastewater: A critical review. J. Hazard. Mater..

[13] Pereira A., Silva L., Laranjeiro C., Lino C., Pena A. (2020). Selected Pharmaceuticals in Different Aquatic Compartments: Part I—Source, Fate and Occurrence. Molecules.

[14] Gao S., Zhao Z., Xu Y., Tian J., Qi H., Lin W., Cui F. (2014). Oxidation of sulfamethoxazole (SMX) by chlorine, ozone and permanganate—A comparative study. J. Hazard. Mater..

[15] Ribeiro A.R., Pedrosa M., Moreira N.F.F., Pereira M.F.R., Silva A.M.T. (2015). Environmental friendly method for urban wastewater monitoring of micropollutants defined in the Directive 2013/39/EU and Decision 2015/495/EU. J. Chromatogr. A.

[16] Gomes J., Costa R., Quinta-Ferreira R.M., Martins R.C. (2017). Application of ozonation for pharmaceuticals and personal care products removal from water. Sci. Total Environ..

[17] Miklos D.B., Remy C., Jekel M., Linden K.G., Drewes J.E., Hübner U. (2018). Evaluation of advanced oxidation processes for water and wastewater treatment—A critical review. Water Res..

[18] European Parliament (2019). European Parliament legislative resolution of 12 February 2019 on the proposal for a regulation of the European Parliament and of the Council on minimum requirements for water reuse (COM(2018)0337-C8-0220/2018-2018/0169(COD)). https://www.europarl.europa.eu/RegData/seance_pleniere/textes_adoptes/provisoire/2019/02-12/0071/P8_TA-PROV(2019)0071_EN.pdf.

[19] Kasprzyk-Hordern B., Ziółek M., Nawrocki J. (2003). Catalytic ozonation and methods of enhancing molecular ozone reactions in water treatment. Appl. Catal. B Environ..

[20] von Gunten U. (2003). Ozonation of drinking water: Part I. Oxidation kinetics and product formation. Water Res..

[21] Bourgin M., Beck B., Boehler M., Borowska E., Fleiner J., Salhi E., Teichler R., von Gunten U., Siegrist H., McArdell C.S. (2018). Evaluation of a full-scale wastewater treatment plant upgraded with ozonation and biological post-treatments: Abatement of micropollutants, formation of transformation products and oxidation by-products. Water Res..

[22] Itzel F., Baetz N., Hohrenk L.L., Gehrmann L., Antakyali D., Schmidt T.C., Tuerk J. (2020). Evaluation of a biological post-treatment after full-scale ozonation at a municipal wastewater treatment plant. Water Res..

[23] Gomes J.F., Frasson D., Pereira J.L., Gonçalves F.J.M., Castro L.M., Quinta-Ferreira R.M., Martins R.C. (2019). Ecotoxicity variation through parabens degradation by single and catalytic ozonation using volcanic rock. Chem. Eng. J..

[24] Carbajo J.B., Petre A.L., Rosal R., Herrera S., Letón P., García-Calvo E., Fernández-Alba A.R., Perdigón-Melón J.A. (2015). Continuous ozonation treatment of ofloxacin: Transformation products, water matrix effect and aquatic toxicity. J. Hazard. Mater..

[25] Asghar A., Lutze H.V., Tuerk J., Schmidt T.C. (2022). Influence of water matrix on the degradation of organic micropollutants by ozone based processes: A review on oxidant scavenging mechanism. J. Hazard. Mater..

[26] Gomes J., Bernardo C., Jesus F., Pereira J.L., Martins R.C. (2022). Ozone Kinetic Studies Assessment for the PPCPs Abatement: Mixtures Relevance. ChemEngineering.

[27] OECD (2006). Test N201: Freshwater Alga and Cyanobacteria, Growth Inhibition Test. OECD Guidelines for the Testing of Chemicals.

[28] OECD (2006). Test N221: *Lemna* sp. Growth Inhibition Test. OECD Guidelines for the Testing of Chemicals.

[29] OECD (2004). Test No. 202: *Daphnia* sp., Acute Immobilisation Test. OECD Guidelines for the Testing of Chemicals.

[30] (2004). Standard Guide for Conducting Daphnia magna Life-cycle Toxicity Tests.

[31] (2016). Soil Quality—Determination of the Toxic Effects of Pollutants on Germination and Early Growth of Higher Plants.

[32] Sahu R.K., Katiyar S., Yadav A.K., Kumar N., Srivastava J. (2008). Toxicity Assessment of Industrial Effluent by Bioassays. Clean–Soil Air Water.

[33] Trautmann N.M., Krasny M.E. (1997). Composting in the Classroom: Scientific Inquiry for High School Students.

[34] Greenberg A., Clesceri L., Eaton A. (1985). Standard Methods for the Examination of Water and Wastewater.

[35] Tay K.S., Rahman N.A., Abas M.R.B. (2010). Kinetic studies of the degradation of parabens in aqueous solution by ozone oxidation. Environ. Chem. Lett..

[36] El Najjar N.H., Touffet A., Deborde M., Journel R., Leitner N.K.V. (2014). Kinetics of paracetamol oxidation by ozone and hydroxyl radicals, formation of transformation products and toxicity. Sep. Purif. Technol..

[37] Domenjoud B., Tatari C., Esplugas S., Baig S. (2011). Ozone-Based Processes Applied to Municipal Secondary Effluents. Ozone Sci. Eng..

[38] Mohapatra D.P., Brar S.K., Tyagi R.D., Picard P., Surampalli R.Y. (2014). Analysis and advanced oxidation treatment of a persistent pharmaceutical compound in wastewater and wastewater sludge-carbamazepine. Sci. Total Environ..

[39] Ikehata K., Naghashkar N.J., El-Din M.G. (2006). Degradation of Aqueous Pharmaceuticals by Ozonation and Advanced Oxidation Processes: A Review. Ozone Sci. Eng..

[40] Tay K.S., Rahman N.A., Abas M.R.B. (2010). Ozonation of parabens in aqueous solution: Kinetics and mechanism of degradation. Chemosphere.

[41] Huber M.M., Canonica S., Park G.-Y., von Gunten U. (2003). Oxidation of pharmaceuticals during ozonation and advanced oxidation processes. Environ. Sci. Technol..

[42] Giri R.R., Ozaki H., Ota S., Takanami R., Taniguchi S. (2010). Degradation of common pharmaceuticals and personal care products in mixed solutions by advanced oxidation techniques. Int. J. Environ. Sci. Technol..

[43] Gomes J., Lincho J., Mazierski P., Miodyńska M., Zaleska-Medynska A., Martins R.C. (2020). Unexpected effect of ozone on the paraben’s mixture degradation using TiO2 supported nanotubes. Sci. Total Environ..

[44] Gomes J.F., Lopes A., Gmurek M., Quinta-Ferreira R.M., Martins R.C. (2019). Study of the influence of the matrix characteristics over the photocatalytic ozonation of parabens using Ag-TiO2. Sci. Total Environ..

[45] Petala A., Frontistis Z., Antonopoulou M., Konstantinou I., Kondarides D.I., Mantzavinos D. (2015). Kinetics of ethyl paraben degradation by simulated solar radiation in the presence of N-doped TiO2 catalysts. Water Res..

[46] Gomes J.F., Leal I., Bednarczyk K., Gmurek M., Stelmachowski M., Diak M., Quinta-Ferreira M.E., Costa R., Quinta-Ferreira R.M., Martins R.C. (2017). Photocatalytic ozonation using doped TiO2 catalysts for the removal of parabens in water. Sci. Total Environ..

[47] Völker J., Stapf M., Miehe U., Wagner M. (2019). Systematic Review of Toxicity Removal by Advanced Wastewater Treatment Technologies via Ozonation and Activated Carbon. Environ. Sci. Technol..

[48] Jesus F., Bernardo C., Martins R.C., Gomes J., Pereira J.L. (2022). Ecotoxicological Consequences of the Abatement of Contaminants of Emerging Concern by Ozonation: Does Mixture Complexity Matter?. Water.

[49] Ferrari B., Mons R., Vollat B., Fraysse B., Paxēaus N., Giudice R.L., Pollio A., Garric J. (2004). Environmental risk assessment of six human pharmaceuticals: Are the current environmental risk assessment procedures sufficient for the protection of the aquatic environment?. Environ. Toxicol. Chem. Int. J..

[50] Xin X., Huang G., Zhang B. (2021). Review of aquatic toxicity of pharmaceuticals and personal care products to algae. J. Hazard. Mater..

[51] Gao F., Li C., Yang Z.-H., Zeng G.-M., Mu J., Liu M., Cui W. (2016). Removal of nutrients, organic matter, and metal from domestic secondary effluent through microalgae cultivation in a membrane photobioreactor. J. Chem. Technol. Biotechnol..

[52] Magdeburg A., Stalter D., Oehlmann J. (2012). Whole effluent toxicity assessment at a wastewater treatment plant upgraded with a full-scale post-ozonation using aquatic key species. Chemosphere.

[53] Coors A., Vollmar P., Sacher F., Thoma A. (2016). Joint Effects of Pharmaceuticals and Chemicals Regulated under REACH in Wastewater Treatment Plant Effluents–Evaluating Concepts for a Risk Assessment by Means of Experimental Scenarios. https://www.umweltbundesamt.de/sites/default/files/medien/1410/publikationen/2017-08-14_texte_61-2017_klaeranlagenablauf.pdf.

[54] Yamamoto H., Tamura I., Hirata Y., Kato J., Kagota K., Katsuki S., Yamamoto A., Kagami Y., Tatarazako N. (2011). Aquatic toxicity and ecological risk assessment of seven parabens: Individual and additive approach. Sci. Total Environ..

[55] Daniel D., Dionísio R., de Alkimin G.D., Nunes B. (2019). Acute and chronic effects of paracetamol exposure on Daphnia magna: How oxidative effects may modulate responses at distinct levels of organization in a model species. Environ. Sci. Pollut. Res..

[56] Carvalho A.R., Pérez-Pereira A.I., Couto C.M.C., Tiritan M.E., Ribeiro C.M.R. (2022). Assessment of effluents quality through ecotoxicological assays: Evaluation of three wastewater treatment plants with different technologies. Environ. Sci. Pollut. Res..

