# Supplementary material for: Ozonation of Selected Pharmaceutical and Personal Care Products in Secondary Effluent—Degradation Kinetics and Environmental Assessment
Source: Toxics. 2022 Dec 8;10(12):765. doi: 10.3390/toxics10120765 (PMC9785417; doi:10.3390/toxics10120765)
Supplement: Supplementary file 1 [file toxics-10-00765-s001.zip › toxics-2091419-supplementary.pdf]

Ozonation of selected pharmaceutical and personal care products in secondary effluent – degradation kinetics and environmental assessment

Fátima Jesus[1], Eva Domingues[2], Carla Bernardo[2], Joana L. Pereira[3], Rui C. Martins[2], João Gomes[2,*]

[1] CESAM - Centre for Environmental and Marine Studies, Department of Environment and Planning, University of Aveiro, Campus de Santiago, 3810-193 Aveiro, Portugal

[2] University of Coimbra, CIEPQPF – Chemical Engineering Processes and Forest Products Research Center, Department of Chemical Engineering, Faculty of Sciences and Technology, Rua Sílvio Lima, 3030-790 Coimbra, Portugal

[3]CESAM - Centre for Environmental and Marine Studies, Department of Biology, University of Aveiro, Campus de Santiago, 3810-193 Aveiro, Portugal

[*] Correspondence author: jgomes@eq.uc.pt

Supplementary material

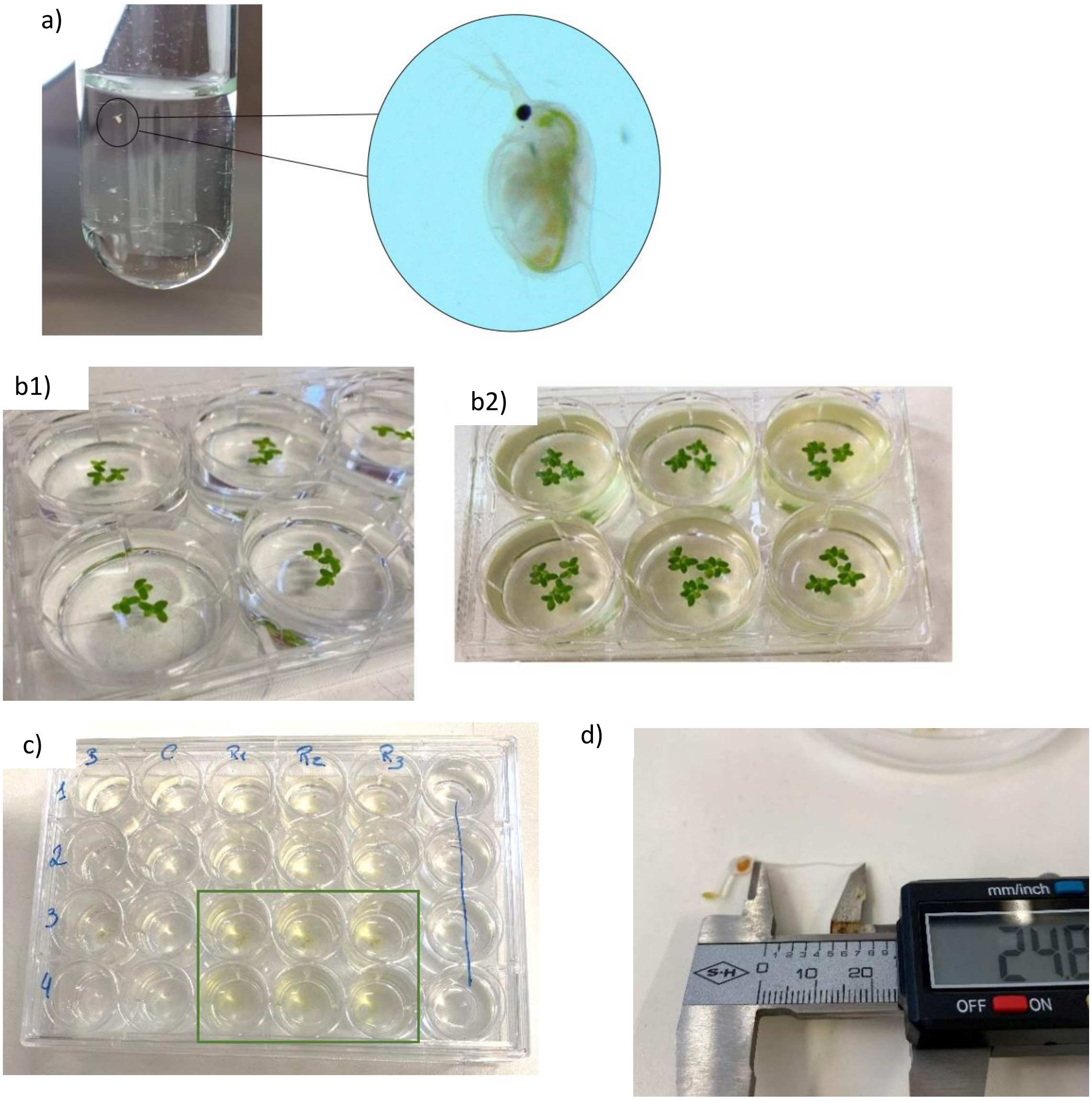

Figure S1: Laboratorial procedures: a) immobilization test with *D. magna*; b) growth inhibition tests with *L. minor* : b1 – start of the test, and b2 – end of the test; c) growth inhibition test with the microalgae *R. subcapitata*; d) germination and growth of *L. sativum* – figure shows the measurement of the length of the root of *L. sativum* with a digital caliper.

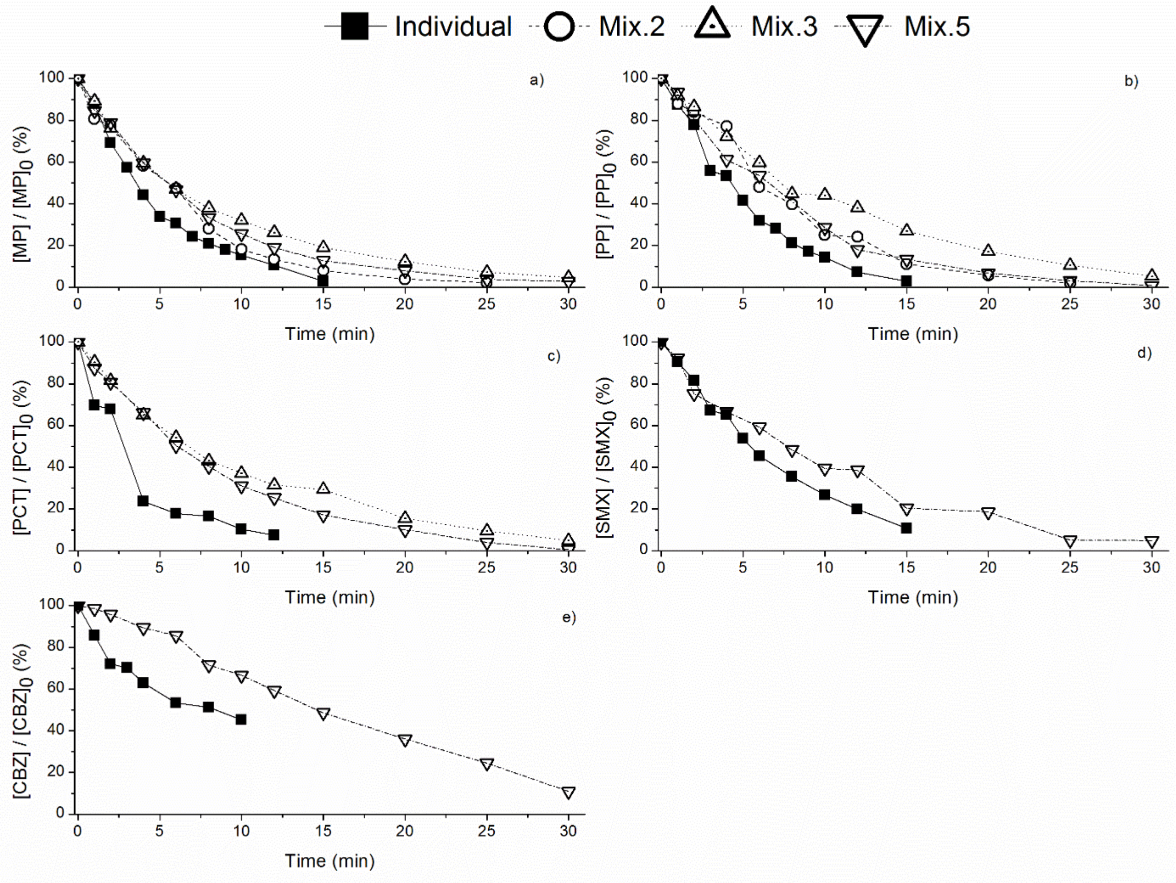

Figure S2: Normalized concentration of: (a) MP; (b) PP; (c) PCT; (d) SMX; (e) CBZ, during ozonation of individual PPCPs and the corresponding mixtures (Mix2, Mix3 and Mix5), throughout the reaction time, using a municipal wastewater as matrix (pH 7), spiked with 1 mg L^{-1} of each PPCP.

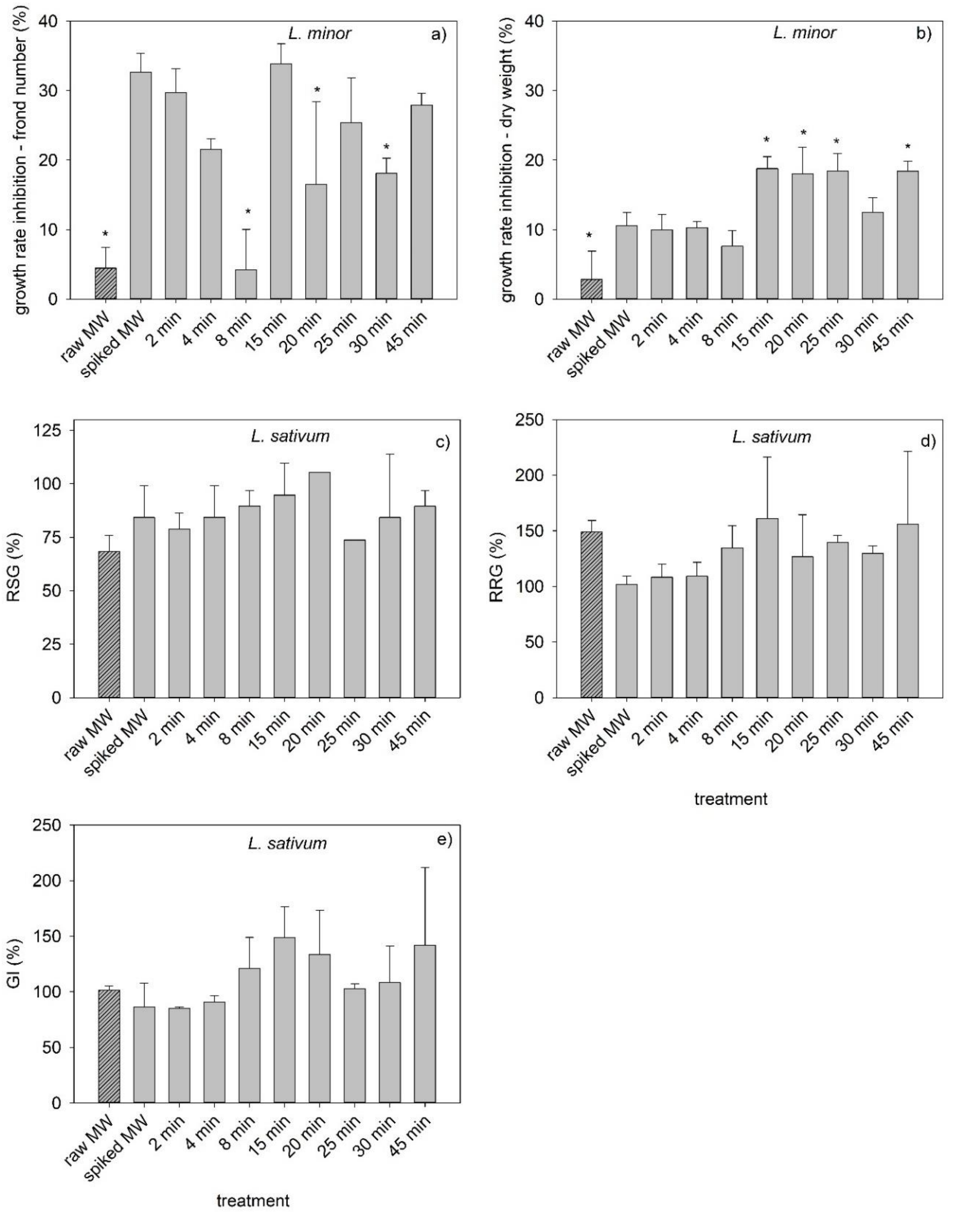

Figure S3: Toxicity response of two biological species exposed to the raw MW, (diagonal lines), and to that MW added five PPCPs (MP, PP, PCT, SMX and CBZ) before ozonation (spiked MW) and after different periods of ozonation. a) growth rate based on the frond number of *L. minor* after 7 d of exposure; b) growth rate based on the dry weight of *L. minor* after 7 d of exposure; c) percentage of RSG of *L. sativum* after 48 h d of exposure; d) percentage of RRG of *L. sativum* after 48 h d of exposure; and e) GI, expressed in percentage, of *L. sativum* after 48 h d of exposure. Bars represent the mean and the error bars represent the standard deviation. Asterisks represents significant differences relative to the untreated spiked MW.

Table S1: Total volume of the nutrient spiking added to each replicate to comply with the standard medium recipe and the corresponding strength of the tested sample (%), for each of the tested species.

Species	Test medium	Total volume of the nutrient spiking added to comply with the test medium recipe	Strength of the tested sample [a]
Raphidocelis subcapitata	Woods Hole MBL [27]	8 μL per replicate	98.2%[b]
Lemna minor	Steinberg [28]	650 μL per replicate	93.5%
Lepidium sativum	Distilled water	---	100%
Daphnia magna	ASTM hard water [30]	800 μL per replicate	92.0%

a) Determined as the volume of sample divided by the final volume of the sample undergoing the ecotoxicological test

b) This accounts for the volume of the nutrient spike (8 μL) and the volume used for microalgae inoculation (10 μL) in each replicate

References

[27] OECD, Test N201: Freshwater Alga and Cyanobacteria, Growth Inhibition Test. OECD Guidelines for the Testing of Chemicals. . 2006, *Organisation for Economic Co-operation and Development*: Paris. p. 25.

[28] OECD, Test N221: *Lemna* sp. Growth Inhibition Test. OECD Guidelines for the Testing of Chemicals. 2006, *Organisation for Economic Co-operation and Development*: Paris. p. 22.

[30] ASTM, Standard Guide for Conducting *Daphnia magna* Life-cycle Toxicity Tests, in ASTM E1193 - 97(2004). 2004, *American Society for Testing and Materials*: Pennsylvania.

Table S2: Comparison of the ecotoxicological response of the test species observed after exposure to the mixture of PPCPs (MP, PP, PCT, SMX and CBZ) at 1 mgL^{-1} each, before (untreated sample) and after ozonation (ozone-treated sample) in ultrapure water and MW. The results regarding tests in ultrapure water were obtained from Jesus et al [48], where the duration of the ozonation reaction was 60 min, whereas the duration of the ozonation reaction in the present study was 45 min. Values highlighted in bold refer to those showing more pronounced differences between the water matrices; in these cases, the values corresponding to the water matrix exhibiting the most favorable response are green shaded.

	Response	Sample treatment	Water matrix	
			Ultrapure water	MW
Raphidocelis subcapitata	Yield inhibition (%)	Untreated	**88%**	**22%**
		Ozone-treated	**83%**	**-72%**
Lemna minor	Yield inhibition (as frond number, %)	Untreated	65%	52%
		Ozone-treated	39%	46%
Lemna minor	Yield inhibition (as dry weight, %)	Untreated	27%	29%
		Ozone-treated	26%	44%
Lepidium sativum	Germination inhibition (%)	Untreated	10%	16%
		Ozone-treated	-6%	11%
Lepidium sativum	Phytotoxicity (%)	Untreated	**69%**	**-2%**
		Ozone-treated	**9%**	**-56%**
Daphnia magna	Immobilization (%)	Untreated	0%	0%
		Ozone-treated	12%	0%

References

[48] Jesus, F., C. Bernardo, R.C. Martins, J. Gomes, and J.L. Pereira. Ecotoxicological Consequences of the Abatement of Contaminants of Emerging Concern by Ozonation: Does Mixture Complexity Matter? *water* **2022**, *14*, 1801.
